# Singlet Fission, Polaron Generation and Intersystem Crossing in Hexaphenyl Film

**DOI:** 10.3390/molecules27165067

**Published:** 2022-08-09

**Authors:** Wenjun Ni, Tianjiao Li, Christian Kloc, Licheng Sun, Gagik G. Gurzadyan

**Affiliations:** 1 School of Sciences, Hangzhou Dianzi University, Hangzhou 310018, China; 2 State Key Laboratory of Fine Chemicals, Institute of Artificial Photosynthesis, Dalian University of Technology, Dalian 116024, China; 3 School of Materials Science & Engineering, Nanyang Technological University, Singapore 639798, Singapore; 4 Center of Artificial Photosynthesis for Solar Fuels, School of Science, Westlake University, Hangzhou 310024, China

**Keywords:** femtosecond spectroscopy, intersystem crossing, singlet exciton fission, time-resolved fluorescence, hexaphenyl film

## Abstract

The ultrafast dynamics of triplet excitons and polarons in hexaphenyl film was investigated by time-resolved fluorescence and femtosecond transient absorption techniques under various excitation photon energies. Two distinct pathways of triplet formation were clearly observed. Long-lived triplet states are populated within 4.5 ps via singlet fission-intersystem crossing, while the short-lived triplet states (1.5 ns) are generated via singlet fission from vibrational electronic states. In the meantime, polarons were formed from hot excitons on a timescale of <30 fs and recombined in ultrafast lifetime (0.37 ps). In addition, the characterization of hexaphenyl film suggests the morphologies of crystal and aggregate to wide applications in organic electronic devices. The present study provides a universally applicable film fabrication in hexaphenyl system towards future singlet fission-based solar cells.

## 1. Introduction

**Figure 1:**
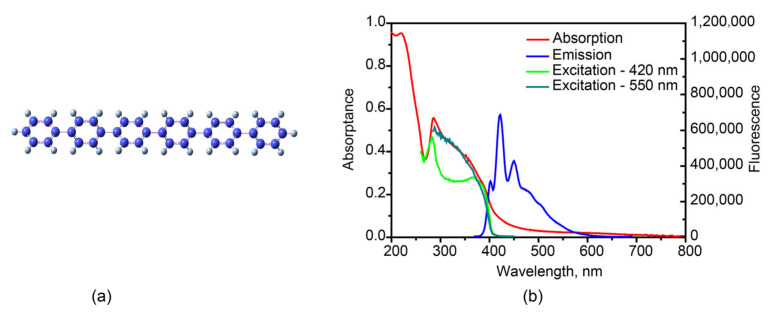
(**a**) Structure of hexaphenyl. (**b**) Steady-state absorption, fluorescence and excitation spectra of hexaphenyl film (λ_exc_ = 350 nm, λ_em_ = 420 and 550 nm).

Organic π-conjugated materials are in the spotlight of interest due to their rich electro-optical properties for wide applications in the field of optoelectronic devices, such as organic light-emitting diodes [1,2,3,4] and solar cells [5]. Among them, short-chain oligomers can be considered as model compounds for unveiling important properties of π electrons in long chain polymers. Para-hexaphenyl, the six phenyls oligomer (Figure 1a), is a promising material, which has been fruitfully applied as efficient blue light emitting diodes [6,7,8,9,10]. It is crucial to explore the fundamental photophysical mechanisms inside this molecule. However, the time-resolved spectroscopic results on triplet formation mechanism are rather rare. The hexaphenyl crystallizes in a monoclinic lattice with the space group *P21/a* and it arranges in a typical herringbone configuration [11,12,13], resulting in good performance on photoelectric devices.

A unique process, singlet fission (SF), was proposed previously to take place in oligomers. It has aroused an increasing interest over the years because of its potential to enhance the efficiency of organic photovoltaics [14,15] and inorganic silicon solar cells [16,17,18,19]. SF is a spin-allowed process where a singlet excited molecule shares its energy with neighboring molecule in its ground state to produce two neighbor-standing triplet excitons. Generally, SF proceeds in ultrafast time scale of femtosecond to picosecond time [20]. SF was observed in organic single crystals [21,22], polymers [23,24], as well as covalently linked dimers [25,26]. Regarding molecular design for SF, the well-studied candidates are limited to polyacene and its derivatives [27]. Further research on this direction will include design of molecular arrays with new kind of π-conjugated molecules. Hence, it is highly desirable to investigate the para-hexaphenyl short-chain oligomers and reveal photophysical relaxation pathways with involvement of upper excited singlet states.

Related ultrafast spectroscopic studies were previously performed on polycrystalline hexaphenyl films [7,8,28] and on para-hexaphenyl-based organic light-emitting devices [29]. The time-resolved photoluminescence unveils the lowest energy emission from H-type hexaphenyl aggregates [28]. By using the pump-probe technique with 500 fs time resolution, the dominant triplet exciton generation mechanism in hexaphenyl is assigned to nongeminate bimolecular recombination of polarons; fusion of singlet excitons and subsequent fission into correlated triplet pairs was clarified [29].

In the present work, we have prepared hexaphenyl film with the thermal vacuum evaporation method and extended the pump-probe detection range down to 350 nm and increased the time resolution to 20–30 fs. Moreover, we have also applied the fluorescence up-conversion setup with a time resolution of 100 fs. These improvements allow direct detection of the triplet transient at 396 nm, and thus, two pathways of triplet state formation via ISC and SF were clearly identified. By tuning the excitation wavelength, we have observed enhanced efficiency of triplet state T_1_ formation via SF and ultrafast polaron formation from higher excited states.

## 2. Materials and Methods

### 2.1. Preparation of the Hexaphenyl Thin Film

The hexaphenyl thin film was prepared by using vacuum evaporation pump device (QHV-Z350C, Pana Instruments) at temperatures of 373–383 K for 20 min; before being installed in the chamber, the quartz substrates were cleaned by acetone in ultrasonic bath for 10 min and soaked in ethanol for 12 h. After that, the quartz substrates were treated with plasma (FEMTO SR CE, Diener) in an O_3_-filled environment for 10 min in order to remove organic impurities and contaminants.

### 2.2. Characterization of the Hexaphenyl Thin Film

XRD data of film were collected using a Rigaku D/Max-2400 X-ray diffractometer in parallel beam geometry employing Cu Kα line focused radiation at 9000 W (45 kV, 200 mA) power and equipped with a position sensitive detector with 10.0 nm radiation entrance slit. Samples were counted on zero background sample holders during the data acquisition. The best counting statistics were achieved by collecting samples using a 0.01° 2θ step scan from 5° to 80° with exposure time of 30 s per step. The structure and morphology of film were investigated by used of field emission scanning electron microscope (FESEM, Nova Nanosem 450). SEM images were measured under 10 kV accelerating voltage and 10 μA current.

### 2.3. Steady-State Absorption and Fluorescence Spectroscopy

UV-vis absorption and fluorescence spectra were obtained by use of a UV-visible spectrophotometer (Cary 100, Agilent) and spectrofluorometer (Fluorolog-3, Horiba Jobin Yvon), respectively.

### 2.4. Time-Resolved Fluorescence Spectroscopy

Fluorescence lifetimes ranging from 10 ps to 20 ns were obtained by time-correlated single photon counting (TCSPC) technique (PicoHarp 300, PicoQuant). By use of deconvolution/fit program (FluoFit, PicoQuant), the time detection limit decreased to 10 ps. The second harmonic of a Titanium-sapphire laser (150 fs, 80 MHz) (Mai Tai DeepSee, Spectra-Physics) at 400 nm was used as the excitation source. Femtosecond fluorescence lifetimes were measured by a fluorescence spectrometer (TRFLS, Newport) with 100 fs resolution in combination with a mode-locked Ti-sapphire laser, the same as mentioned in TCSPC. The femtosecond laser system generated light pulses at 800 nm of duration 150 fs at a repetition rate 80 MHz, average power of 2.9 W. The frequency of the laser pulse was doubled with a BBO crystal and served for excitation (pump). The emitted fluorescence was focused into a BBO crystal together with the gate beam (800 nm) to create the up-converted signal at the sum-frequency generation. The overall time resolution of the setup was 100 fs. Fluorescent images were acquired with confocal microscopy Olympus FV1000.

### 2.5. Transient Absorption Spectroscopy

The transient absorption (TA) spectra were measured by use of a home-made pump-probe setup, described in detail previously [26,30]. The output of a mode-locked Ti-sapphire amplified laser system (Spitfire Ace, Spectra-Physics), with a wavelength of 800 nm, pulse-width of 35 fs, repetition rate of 1 kHz and average power of 4 W, was split into two beams (10:1). Pump beam in the range of 240–2400 nm is acquired by use of an Optical Parametric Amplifier (TOPAS, Light Conversion). The pump pulse duration was 42 fs (measured from the risetime of TA kinetics for Rhodamine 6G). Deconvolution fit process allows obtaining three times higher resolution than the pulse width of the laser, i.e., 20–30 fs [31,32,33]. Part of 800 nm laser pulse was focused in a 3 mm thick, rotated CaF_2_ plate to produce a white light continuum (WLC), which was used as a probe beam ranging from 350 to 850 nm. WLC propagated through the sample and directed into the diode array attached to spectrograph (Jobin Yvon CP140) with detection range 250–850 nm (Entwicklungsbuero Stresing, Germany).

The entire setup was controlled by a PC with the help of LabView software (National Instruments). All measurements were performed at room temperature under aerated conditions.

The experimental data were fitted to a multiexponential decay function convoluted with the instrument response function *B* (*t* − *t_0_*) centered at *t*_0_:

(1) $$\Delta A(t)=\int_{0}^{\infty }\left(\Delta A_{0}+\sum_{i=1}^{n}\Delta A_{i}\exp\left(-\frac{t^{\prime }}{t_{i}}\right)\right)\cdot B(t-t^{\prime }-t_{0})dt^{\prime }$$

where Δ*A(t)* is the difference absorption at time *t*, Δ*A_i_* is the amplitude of the component with lifetime *τ_i_* and Δ*A_0_* is the offset due to long-living species. The instrument response function was modeled by a Gaussian with a variable Full Width Half Maximum (FWHM).

### 2.6. Global Analysis

Global lifetime analysis of time-resolved experimental results was performed by Glotaran software [34]. The Decay Associated Spectra (DAS) allow separating several overlapping spectra in parallel model, whereas the Evolution Associated Spectra (EAS) shows the global fit results in a sequential model. The Species Associated Spectra (SAS) use to separate the process which is a combination of the parallel and sequential model and involves branching, back-reactions or multiple compartment excitation.

## 3. Results and Discussion

### 3.1. Steady-State Absorption and Fluorescence Spectra of Hexaphenyl Film

Steady-state absorption, fluorescence and fluorescence excitation spectra of hexaphenyl film were measured, presented in Figure 1b. The film absorption spectrum (Figure 1b red curve) shows two broad bands between 200 and 480 nm with the maximum at 285 and 220 nm. Compared to hexaphenyl solution (Figure S1), the absorption spectra of film are broad and unstructured.

The spectroscopic feature of film ascribes to its conformational disorder (inhomogeneous broadening), i.e., the variety of oligomer geometrical arrangements due to the torsional degree of freedom of adjacent rings. Inhomogeneous broadening of the optical absorption in conjugated oligomers is attributed to the conjugation length distribution [35]. The fluorescence spectrum exhibits a broad band with three maxima at 402, 422 and 449 nm. These vibronic structures are ascribed to a strong electron-vibronic coupling that favors the formation of polaron-excitons during the optical excitation of the molecules, in agreement with previous studies on luminescent polymers and oligomers [36,37].

Surprisingly, the fluorescence excitation spectra with various probe wavelengths at λ = 420 and 550 nm are entirely different (Figure 1b). At 550 nm, the excitation spectra fit very well with absorption. Understandably, it should follow Kasha–Vavilov’s rule [38,39]. However, at 420 nm there is a strong deviation between 280 and 380 nm, indicating that fluorescence emission at 420 and 550 nm is originating from different species of monomer and aggregate, respectively. Another possibility is that the fluorescence is affected by reabsorption [40,41]. In order to clarify the origin of the emission species, the photoluminescence spectra of hexaphenyl in cyclohexane and single crystal were recorded (Figure S2). To follow the transition assignments, Gaussian multipeak analysis was performed for both film and crystal (Figure S3), and the resulting maxima were recorded in Table S1. In the film, constant energy spacing is 0.17 eV (as obtained from the fluorescence spectrum), corresponding to the stretching vibration of the carbon backbone [42]. The crystal emission shows three maxima of 2.60, 2.77 and 2.89 eV, which exist in the film as well. As known, the hexaphenyl crystal is slip-stacked and aligned in a face-to-tail herringbone arrangement (J-aggregations). Similar to crystal, J-aggregated morphology of the film contributes to the emission between 425 and 600 nm. Furthermore, hexaphenyl monomer in solution shows one broad band at shorter wavelength 325–450 nm, which is not observed in hexaphenyl film.

### 3.2. Various Emission Species in Hexaphenyl Film

**Figure 2:**
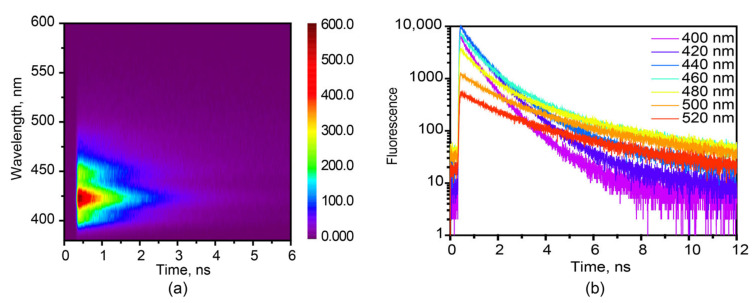
(**a**) Fluorescence TCSPC map of the hexaphenyl film, λ_exc_ = 360 nm. (**b**) Fluorescence kinetics of hexaphenyl film at various probe wavelengths, λ_exc_ = 360 nm.

The fluorescence map and kinetics of hexaphenyl film in 380–600 nm are recorded in Figure 2 at λ_exc_ = 360 nm, by use of TCSPC technique. The fluorescence lifetime of hexaphenyl in hexane is presented in Figure S4a with single-exponential decay τ = 0.77 ns. The fitting results of film fluorescence decay kinetics are shown in the Table S2: τ_1_ = 0.24–0.39 ns (A_1_ = 0.42–0.67), τ_2_ = 0.64–1.05 ns (A_2_ = 0.30–0.43) and τ_3_ = 1.6–3.9 ns (A_3_ = 0.05–0.18). In order to properly assign the origin of each time component, the map data were globally fit, resulting in τ_1_ = 0.30, τ_2_ = 0.85 and τ_3_ = 3.6 ns. Decay-associated spectra (DAS) are shown in Figure S5.

The shorter lifetimes τ_1_ = 0.3 and τ_1_ = 0.85 ns are due to the heterogeneity in organic film. They shared similar DAS, and therefore, we ascribe to the aggregate state in slightly different environments in film. These two times are in line with reported fluorescence lifetime τ = 279 ps from aggregated hexaphenyl film [28]. The shorter lifetimes τ_1_ = 0.3 ns and τ_2_ = 0.85 ns are due to the heterogeneity of organic film. They shared similar DAS, and therefore, we attribute them to the aggregated states in slightly different environment.

The longest lifetime, τ_3_ = 3.6 ns, we assign to the fluorescence from crystalline morphology in film. It agrees well with the fluorescence lifetime of 2.6 ns obtained for hexaphenyl single crystal (Figure S4b). The XRD data of hexaphenyl film reveal the sharp peak in the same position of crystal (Figure S6), in agreement with previous publications [13,43], suggesting the existence of crystalline morphology in film. Moreover, fluorescence confocal microscopy was performed (Figure S7). The resulting fluorescence image shows several types of fluorescing ingredients on the surface with lifetimes ranging from 0.13 to 1.9 ns, indicating the heterogeneity of the film. Besides, the SEM image (Figure S8) exhibits clearly both aggregated and crystalline morphologies. To sum up, the obtained fluorescence lifetimes depend on probe wavelength and are explained by the inhomogeneous broadening. This heterogeneous phenomenon can be attributed to the existence of different lattice locations where the molecules experience various local electric and magnetic fields.

Furthermore, the up-conversion technique with higher time-resolution was applied in order to resolve the ultrafast fluorescence in hexaphenyl film at λ_exc_ = 400 nm (Figure S9). It gives two decays: τ_1_ = 22–35 ps (A_1_ = 0.22–0.25) and τ_2_ = 220–300 ps (A_2_ = 0.69–0.74) (Table S3). They accord to the aggregates’ lifetime [28].

### 3.3. Transient Absorption Spectra of Hexaphenyl Film

**Figure 3:**
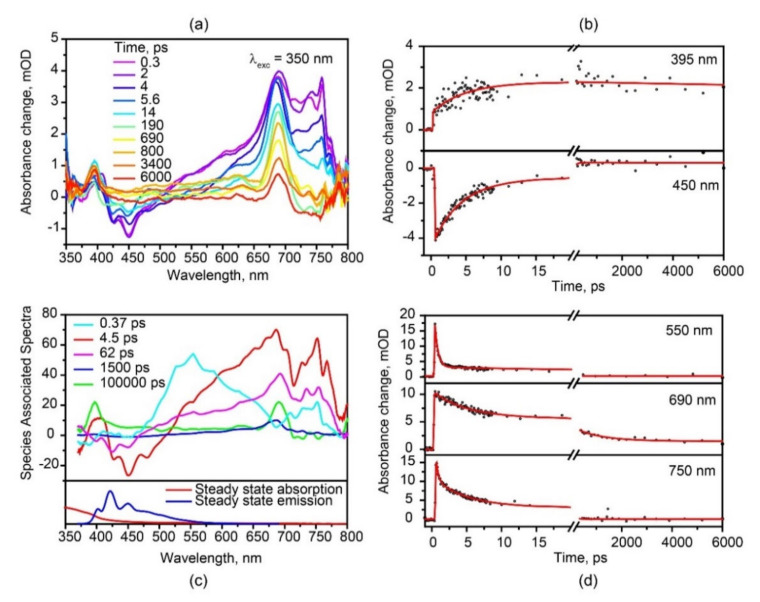
Transient absorption spectra (**a**) and Species Associated Spectra by global fit (**c**) of hexaphenyl film; decay kinetics (**b**,**d**) at various probe wavelengths. λ_exc_ = 350 nm.

Femtosecond transient absorption (TA) spectra of hexaphenyl film at λ_exc_ = 350 nm are shown in Figure 3a. The TA kinetics at different probe wavelengths are shown in Figure 3b,d; fit data at λ_exc_ = 350 nm are summarized in Table 1. The TA spectra display three excited state absorption (ESA) bands at 397, 687 and 752 nm and a broad unstructured band between 510 and 650 nm. The negative band at 410–490 nm is a mirror image of the steady-state fluorescence spectrum. Therefore, this band is assigned to stimulated emission (SE), which exhibits the two time constants of 4.5 and 89 ps, in agreement with the above fluorescence data. Note that SE is distorted by positive transients at longer delay times. It should be mentioned that we did not observe a ground state bleaching (GSB) signal because it overlaps with the strong ESA band at 397 nm.

The positive ESA at 752 nm is due to singlet-singlet absorption, in line with the TA data of [28]. This band decays tri-exponentially with 0.3, 4.3 and 84 ps; the latter two lifetimes are similar to SE decay (4.5 and 89 ps). The ultrafast time of 0.3 ps we ascribe to polaron formation [29]. When the lattice phonons are strongly coupled to the charge carriers in the molecular crystal, it results in the formation of localized polaron [44]. Polarons of several hundreds of femtoseconds with and without an applied external field were previously observed in hexaphenyl film by Zenz et al. [29].

### 3.4. Two Pathways of Triplet Formation

Furthermore, in our TA spectra, there are two longer lived ESA species at 397 and 687 nm. The 687 nm transient band we assign to triplet-triplet absorption, in agreement with previous publications of hexaphenyl [6,28,29] and oligothiophene films [45,46]. In our film, this triplet state forms instantaneously (<30 fs, i.e., within time resolution) and decays with two time constants: 1 ns and >>100 ns. We conclude that ultrafast formation of triplet excitons is due to singlet fission in hexaphenyl aggregates. With 350 nm (3.54 eV) excitation, we are populating the higher vibrational levels of S_1_. Thus, direct SF proceeds from the upper vibrational levels of the S_1_ within <30 fs. Note that usually the triplet states live much longer, i.e., micro- and milliseconds. Our triplets are relatively short-lived due to efficient triplet-triplet annihilation between nearby triplet excitons formed via SF. The 687 nm TA band is overlapped with strong singlet-singlet and polaron transient spectra; therefore, global analysis is made in order to separate the time constants. Furthermore, in Figure 3a, the “clean” ESA band at 397 nm has a long-lived component (>100 ns); we assign this long-lived transient to triplet-triplet absorption as well. This triplet band rises with two time constants: ultrafast (<30 fs) and 4.5 ps, which is indicative of two pathways of triplet formation, i.e., via SF and intersystem crossing (ISC), respectively (see discussion below)

In order to clarify the full photophysical pathways, the fs-TA data at λ_exc_ = 350 nm were analyzed by Glotaran software. We have applied the target model where two pathways of triplet formation are involved, i.e., one from the lowest singlet state and another from upper vibrational states of S_1_. It includes also the processes of polaron formation and energy transfer from the hot S_1_ state to the aggregate S_1_ state (Figure S10). Resulting Species Associated Spectra (SAS), with the five components of 0.37, 4.5, 62 and 1500 ps and >100 ns, are presented in Figure 3c. These time constants are assigned to polaron, two aggregates S_1_, short-lived states and long-lived triplet states, respectively. The broad spectrum of ultrashort-lived polaron is correlated with the previous publication [29]. The SAS of monomer and aggregate singlet reveal negative SE transients. They both correspond to various bands of the steady-state fluorescence spectrum. This is indicative of presence of different emissive species.

Obviously, triplet transient(s) should be the same independent on how they are formed, whether directly via ISC or via SF. Global analysis clearly reveals that the spectra of short-lived and long-lived triplets display the same maxima, both at 397 and 687 nm, indicating the same origin of these bands. Thus, triplets formed directly via ISC or via SF show the same TA. However, their formation and decay kinetics are quite different. In our case, triplet states formed via ISC have a 4.5 ps rise time, which corresponds to the singlet decay τ = 4.3 ps; the lifetime of *this [single]* triplet state is long (>100 ns). In contrary, triplet states via SF are formed instantaneously (rise time <30 fs); *this pair* of triplet states has a short lifetime, τ = 1.5 ns (because of efficient triplet-triplet annihilation).

**Figure 4:**
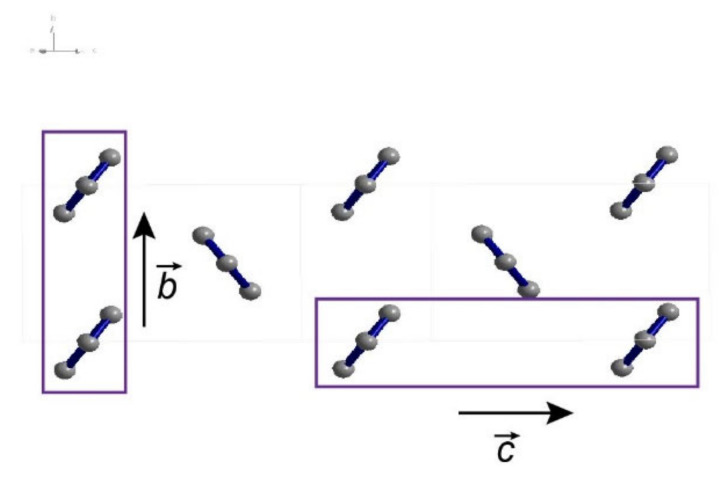
Correlation of packing scheme along different crystal axes. Schematics of hexaphenyl stacking patterns along the *b*- and *c*-axes.

First-principles TD-DFT calculations based on b31yp/6-31g(d) were performed and the resulting the energy levels of the singlet and triplet states were obtained at the optimized excited state geometry. The first excited singlet state is located at 3.00 eV (Figure S11), in agreement with previous reports [47]. The lowest triplet state T_1_ energy optimized by the same method is 1.04 eV (Figure S12). Thus, it possesses the relationship 2E(T_1_) < E(S_1_) required for singlet fission. The schematic stacking patterns of hexaphenyl single crystal along the *b*- and *c*-axes is shown in Figure 4. The hexaphenyl molecules are slip-stacked and aligned in a face-to-tail herringbone arrangement. In organic semiconductors, the packing configuration of molecules influences strongly the SF efficiency: slip-stacking favors delocalization of excitation and facilitate exciton fission [48]. Hence, in hexaphenyl film, triplets can be generated via SF in such optimal packings.

In previous publications, the Wasielewski group [49] has reported in the solution of anthracene derivatives, spin-orbit-induced intersystem crossing (SO-ISC) contributes to 53% of triplet formation and SF attributes to the remaining 47%. In this system, SO-ISC originates from individual molecule and SF results from aggregates [49]. In crystalline tetracene [50], the switching between singlet fission and intersystem crossing was manipulated by suppressing intermolecular coupling and lowering the temperature. Moreover, they proposed that the spin dephasing time of triplet pair generated by SF is 40 ns and the lifetime of triplets formed in isolated molecule via ISC is 100 times longer [50]. These observations are in line with our short-lived triplet states generated via SF and long-lived triplets formed via ISC. In the case of tetrachlorinated phenazinothiadiazole [51], the formation mechanism of triplet state depends on concentration. At higher concentrations, TA spectra show the occurrence of a second triplet forming process besides ISC, which is SF, while at lower concentrations, there is only ISC [51]. In pentacene derivatives [52], the introduction of a radical substituent greatly accelerated intersystem crossing and notably enhanced singlet fission [52]. Moreover, in nonconjugated covalent pentacene dimer SF dominates in a dimer, while ISC occurs in its remaining monomeric moiety with much lower efficiency [53]. Note that the triplet states of the abovementioned molecules are located very far away from the singlet state (E(S_1_) ≈ 2E(T_1_)); still, both ISC and SF may happen under this condition. Therefore, we believe that both ISC and SF exist in hexaphenyl film.

We explained the mechanism of ISC in hexaphenyl film in the term of singlet fission intersystem crossing (SF-ISC), which was proposed previously for anthanthrene derivatives [49]. The triplet states were populated efficiently with quantum yield of 30% despite the large S_1_-T_1_ energy gap (>1 eV) and the lack of carbonyl groups or heavy atoms. This mechanism was based on previous studies in both polyacenes and terrylenediiimides [53,54,55,56,57]: singlet fission can proceed with formation of an initial spin-correlated triplet pair state ^1^(T_1_T_1_) that can intersystem cross to a quintet spin state ^5^(T_1_T_1_), which further separated to form two T_1_ states. The quintet state is formed with its m_s_ = 0 sublevel greatly over-populated, so that this initial non-Boltzmann spin population is carried over to the m_s_ = 0 sublevel of the T_1_ states. This population of T_1_ is labeled as SF-ISC. They attribute the appearance of the SF-ISC triplet to a change in the electronic coupling among the aggregated molecules. Similarly, in the case of hexaphenyl film, we identified the heterogeneity of the film from various herringbone and parallel orientations of hexaphenyl due to a change in the dipolar coupling. Therefore, the SF channel in crystalline hexaphenyls result in the population of ^5^(T_1_T_1_). Another channel of triplet formation will involve SF-ISC mechanism with lifetime of 4.5 ps, which corresponds to the singlet state lifetime.

As already mentioned, our film contains aggregated and crystalline morphologies; ISC proceeds in aggregated hexaphenyl and resulting triplets are long-lived (>100 ns). While SF takes place in this film, the generated nearby triplets live much shorter (1.5 ns) due to efficient triplet-triplet annihilation.

Microscopically, both SF and ISC are electronically nonadiabatic processes. Since SF is spin allowed and ISC is spin forbidden, the nonadiabatic coupling constant (and therefore, transfer rate) is usually much higher for SF than for ISC. Direct SF, notably if it is mediated by a conical intersection as for rubrene crystal [58], can occur on a sub 100 fs timescale, e.g., theoretical simulations for pentacene [59] and rubrene [60]. As for ISC, it is well known that it can become faster in molecular crystals/films, due to favorable geometric constraints, which may lead to (i) enhancement of through space/bond couplings and (ii) alignment of electronic and vibrational levels causing vibration-mediated coherent effects [61,62]. Therefore, triplet formation will occur both via ISC and SF. Further concerted experimental and theoretical efforts are necessary to clarify microscopic origin and pathways of the triplet formation in hexaphenyl films. For pentacene, for example, such a study exists [53].

**Figure 5:**
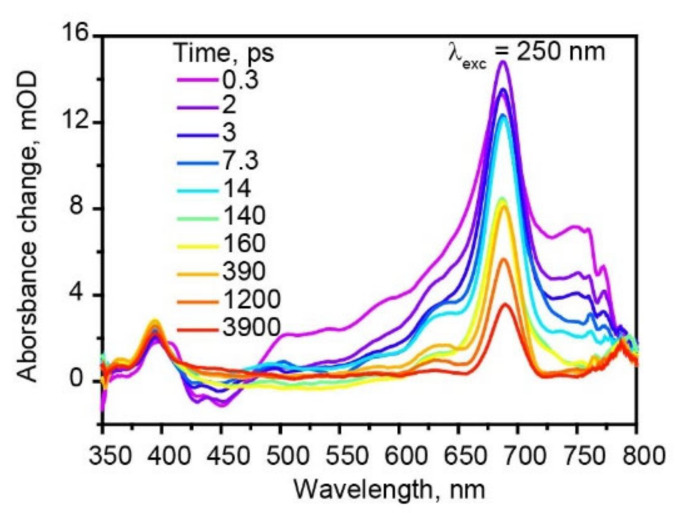
Femtosecond transient absorption spectra of hexaphenyl film at λ_exc_ = 250 nm.

Furthermore, we have excited hexaphenyl film with 250 nm (Figure 5), thus populating higher-lying electronic states. The TA kinetics at different probe wavelengths are shown in Figure S13; fit data at λ_exc_ = 250 nm are summarized in Table S4. Two triplet state absorption bands are more pronounced compared with 350 nm excitation. From the comparison of the kinetics and the amplitudes of two triplet absorption bands at 398 and 689 nm, we found that at λ_exc_ = 350 nm 61% triplet excitons are formed via ISC and 26% via SF. However, at λ_exc_ = 250 nm, 47% triplet excitons are formed via ISC, while 39% via SF. Thus, larger excitation photon energies lead to more efficient SF.

## 4. Conclusions

We have studied the excited state dynamics of hexaphenyl film with a femtosecond pump-probe and fluorescence spectroscopy. In this heterogeneous film we found different emission species: aggregated and crystalline parts. From transient absorption, an ultrafast (30 fs) singlet fission directly from higher vibrational levels was observed. Another route of triplet state formation is intersystem crossing from the lowest singlet state within 4.5 ps. Moreover, an ultrafast polaron generation was also detected at different excitation conditions. DFT calculations support our experimental data and conclusions.

Our film with coexistence of aggregated and crystalline morphologies could exhibit efficient singlet fission. The present results give a new insight into the excited state dynamics in hexaphenyl film, further contributing to the understanding of singlet fission in organic crystals and films. Moreover, the simple-fabricated commercially available hexaphenyl film is highly promising material for organic photovoltaic applications.

## Figures and Tables

**Table 1:** Lifetimes obtained from Fit/Deconvolution of TA dynamics for the hexaphenyl film at λ_exc_ = 350 nm; f = fixed.

λ_exc_, nm	λ_probe_, nm	τ_1_, ps	A_1_	τ_2_, ps	A_2_	τ_3_, ps	A_3_	τ_4_, ps	A_4_
350	395	4.5	−0.61					100,000 f	1
	450	4.5	−0.75	89	−0.25			100,000 f	1
	550	0.45	0.85	62	0.15				
	690	4.6	0.41	65	0.18	1000	0.26	100,000 f	0.15
	750	0.30	0.43	4.3	0.37	84	0.20		

## Data Availability

Not applicable.

## Supplementary Materials

The following supporting information can be downloaded at: https://www.mdpi.com/article/10.3390/molecules27165067/s1, Figure S1: Steady state absorption and fluorescence spectra of hexaphenyl in hexane (λ_exc_ = 300 nm); Figure S2: Fluorescence of hexaphenyl solution (λ_exc_ = 300 nm), film (λ_exc_ = 350 nm) and single crystal (λ_exc_ = 350 nm); Figure S3: Gaussian fit for steady state fluorescence spectra of (a) hexaphenyl film and (b) single crystal; Figure S4: Fluorescence decay kinetics of the (a) hexaphenyl solution and (b) single crystal λ_exc_ = 267 nm at various probe wavelengths; Figure S5: Global fit spectra of TCSPC results for hexaphenyl film at λ_exc_ = 360 nm; Figure S6: X-ray diffraction (XRD) patterns of hexaphenyl film and single crystal presented on the same scale; Figure S7: Fluorescence lifetime imaging acquired with confocal microscopy. Range size (a): 1 × 1 μm, (b): 10 × 10 μm; Figure S8: Scanning electron microscope (SEM) of hexaphenyl film; Figure S9: Up-conversion fluorescence decay kinetics of hexaphenyl film at λ_exc_ = 400 nm; Figure S10: Global fit target model (a) and kinetics (b) of Species Associated Spectra; Figure S11: TD-DFT/(b3lyp/6-31g(d)) calculation of excited singlet state energies of hexaphenyl isolated molecule at S_1_ geometry; Figure S12: TD-DFT/(b3lyp/6-31g(d)) calculation of excited triplet state energies of hexaphenyl isolated molecule at T_1_ geometry; Figure S13: Decay kinetics of the hexaphenyl film λ_exc_ = 250 nm at different wavelengths; Table S1: Gaussian multipeak fit maxima of the steady state fluorescence spectra of hexaphenyl film and single crystal; Table S2: Fluorescence decay kinetics of hexaphenyl film, solution and single crystal at various emission wavelengths (TCSPC data); Table S3: Fluorescence decay kinetics of hexaphenyl film at various emission wavelengths (up-conversion data). τ_3_ is fixed to respective TCSPC data; Table S4: Lifetimes obtained from fit/deconvolution of TA spectra for the hexaphenyl film at λ_exc_ = 250 nm; f = fixed.

## Sample Availability

Samples of the compounds are not available from the authors.
