# Supplementary material for: Singlet Fission, Polaron Generation and Intersystem Crossing in Hexaphenyl Film
Source: Molecules. 2022 Aug 9;27(16):5067. doi: 10.3390/molecules27165067 (PMC9412266; doi:10.3390/molecules27165067)
Supplement: Supplementary file 1 [file molecules-27-05067-s001.zip › molecules-1849371-supplementary.pdf]

Supplementary Materials

for

Singlet Fission, Polaron Generation and Intersystem Crossing in Hexaphenyl Film

Wenjun Ni [1], Tianjiao Li [2], Christian Kloc [3], Licheng Sun [2,4] and Gagik G. Gurzadyan [2,*]

[1]School of Sciences, Hangzhou Dianzi University, Hangzhou 310018, China

[2] State Key Laboratory of Fine Chemicals, Institute of Artificial Photosynthesis, Dalian University of Technology, Dalian 116024, China

[3] School of Materials Science & Engineering, Nanyang Technological University, Singapore 639798, Singapore

[4] Center of Artificial Photosynthesis for Solar Fuels, School of Science, Westlake University, Hangzhou 310024, China

Correspondence: gurzadyan@dlut.edu.cn; Tel: +86-411-8498-6489

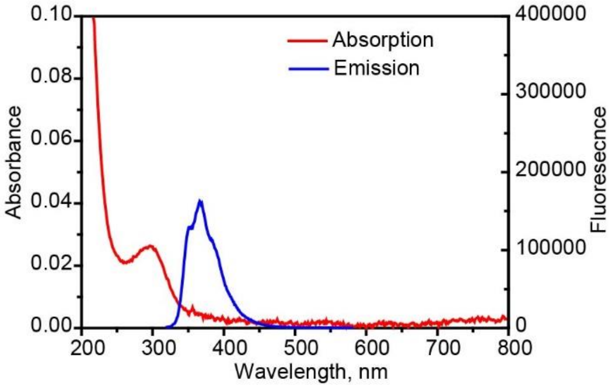

Figure S1. Steady state absorption and fluorescence spectra of hexaphenyl in hexane (λ_{exc} = 300 nm).

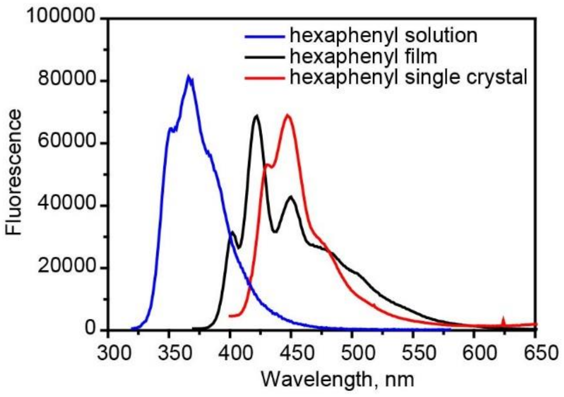

Figure S2. Fluorescence of hexaphenyl solution (λ_{exc} = 300 nm), film (λ_{exc} = 350 nm) and single crystal (λ_{exc} = 350 nm).

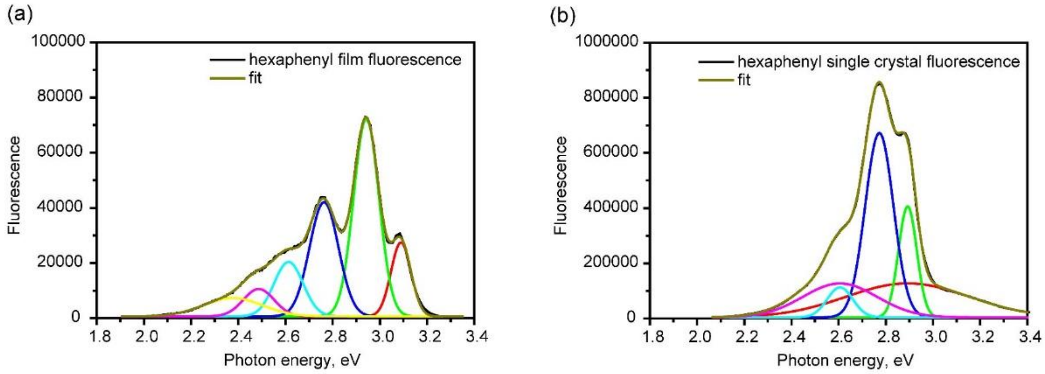

Figure S3. Gaussian fit for steady state fluorescence spectra of (a) hexaphenyl film and (b) single crystal.

Table S1. Gaussian multipeak fit maxima of the steady state fluorescence spectra of hexaphenyl film and single crystal.

System	Maxima of emission bands [eV]					
Film	2.37	2.49	2.61	2.76	2.94	3.11
Single crystal			2.60	2.77	2.89	

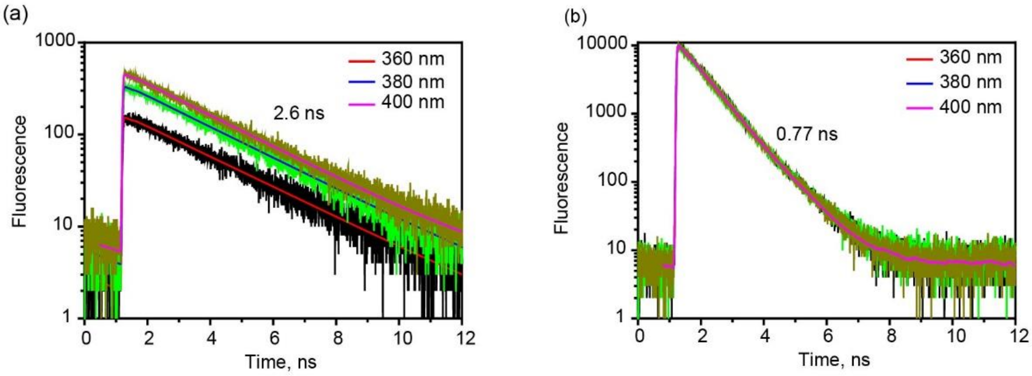

Figure S4. Fluorescence decay kinetics of the (a) hexaphenyl crystal and (b) in hexane λ_{exc} = 267 nm at various probe wavelengths.

Table S2. Fluorescence decay kinetics of hexaphenyl film, solution and single crystal at various emission wavelengths (TCSPC data).

λ_{exc}, nm	λ_{probe}, nm	τ_1, ns	A_1	τ_2, ns	A_2	τ_3, ns	A_3
Film 360	400	0.24	0.62	0.76	0.35	1.6	0.03
	420	0.30	0.70	0.70	0.30	1.9	0.01
	440	0.32	0.67	0.73	0.32	2.9	0.01
	460	0.32	0.60	0.91	0.34	3.9	0.06
	480	0.30	0.57	0.93	0.32	3.4	0.11
	500	0.27	0.44	1.02	0.40	3.4	0.16
	520	0.26	0.42	1.05	0.40	3.5	0.18
Crystal 267	360					2.6	1
	380					2.6	1
	400					2.6	1
Solution 267	360			0.77	1		
	380			0.77	1		
	400			0.77	1		

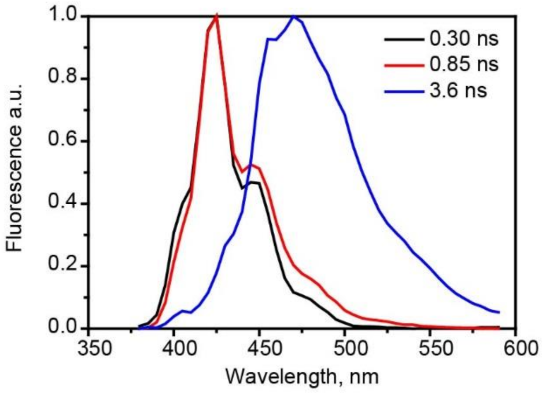

Figure S5. Global fit spectra of TCSPC results for hexaphenyl film at λ_{exc} = 360 nm.

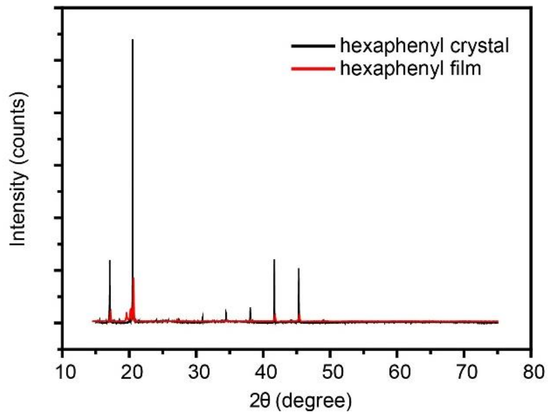

Figure S6. X-ray diffraction (XRD) patterns of hexaphenyl film and single crystal presented on the same scale.

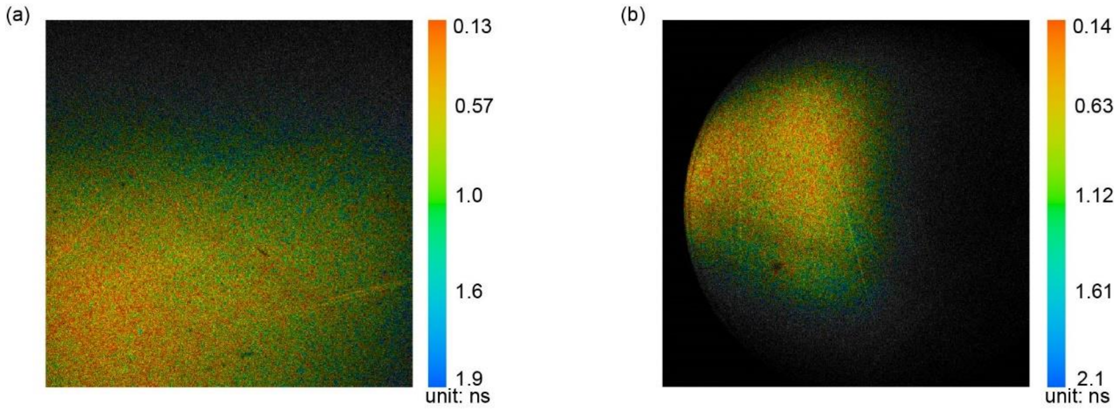

Figure S7. Fluorescence lifetime imaging acquired with confocal microscopy. Range size (a): 1*1 μm, (b): 10*10 μm.

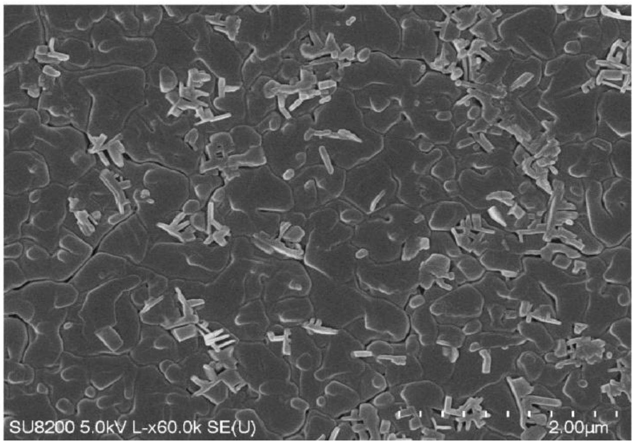

Figure S8. Scanning electron microscope (SEM) of hexaphenyl film.

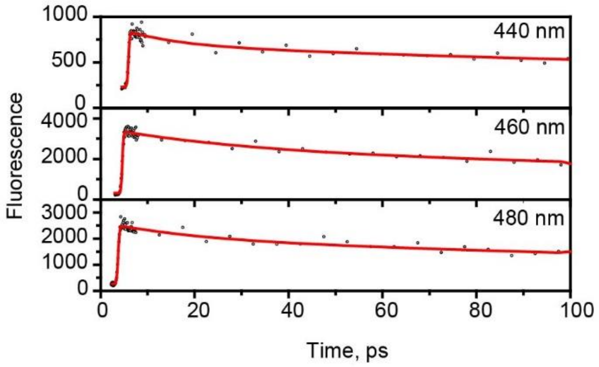

Figure S9. Up conversion fluorescence decay kinetics of hexaphenyl film at λ_{exc} = 400 nm.

Table S3. Fluorescence decay kinetics of hexaphenyl film at various emission wavelengths (up-conversion data). τ_3 is fixed to respective TCSPC data.

λ_{exc}, nm	λ_{probe}, nm	τ_1, ps	A_1	τ_2, ps	A_2	τ_3, ps	A_3
400	440	29	0.24	250	0.74	4000^f	0.02
	460	35	0.25	300	0.69	4000^f	0.06
	490	27	0.22	270	0.70	4000^f	0.08

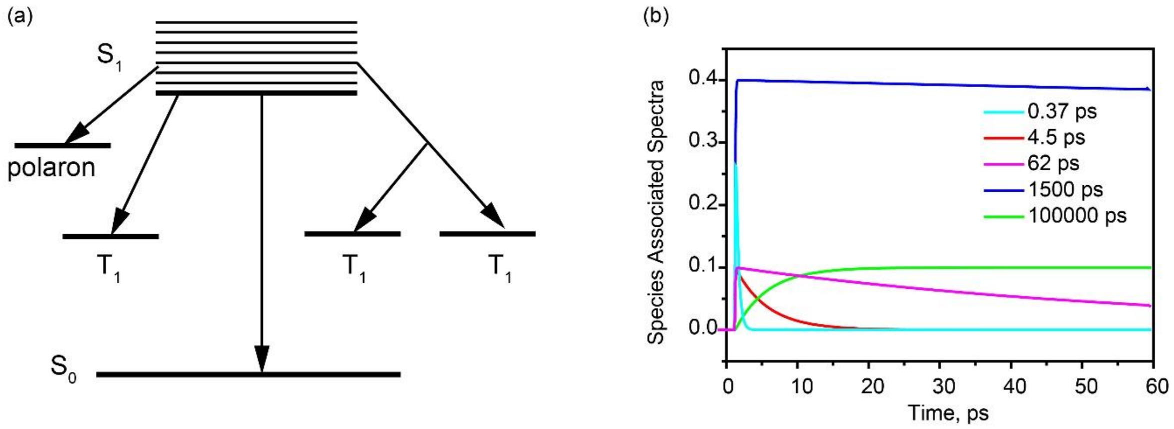

Figure S10. Global fit target model (a) and kinetics (b) of Species Associated Spectra.

```
Excitation energies and oscillator strengths:

Excited State   1:      Singlet-A      3.0022 eV  412.97 nm  f=2.6586  <S**2>=0.000
    121 ->122         0.70148
This state for optimization and/or second-order correction.
Total Energy, E(TD-HF/TD-KS) =  -1386.01198264
Copying the excited state density for this state as the 1-particle RhoCI density.

Excited State   2:      Singlet-A      3.7356 eV  331.90 nm  f=0.0000  <S**2>=0.000
    120 ->122        -0.57289
    121 ->123        -0.39899

Excited State   3:      Singlet-A      3.9383 eV  314.81 nm  f=0.0000  <S**2>=0.000
    120 ->122         0.40184
    121 ->123        -0.57400
```

Figure S11. TD-DFT/(b3lyp/6-31g(d)) calculation of excited singlet state energies of hexaphenyl isolated molecule at S_1 geometry.

```
Excitation energies and oscillator strengths:

 Excited State   1:  3.007-A     0.1408 eV 8803.98 nm f=0.0001 <S**2>=2.011
    122A ->123A       0.92075
    122A ->131A       0.29118
    110B ->121B       0.10256
    112B ->121B      -0.30610
    120B ->121B       0.95325
    122A <-123A       0.64240
    122A <-131A       0.24087
    112B <-121B      -0.24646
    120B <-121B       0.64186
 This state for optimization and/or second-order correction.
 Total Energy, E(TD-HF/TD-KS) = -1387.41406581
 Copying the excited state density for this state as the 1-particle RhoCI density.

 Excited State   2:  3.027-A     1.0392 eV 1193.05 nm f=0.0000 <S**2>=2.041
    122A ->125A       0.75914
    122A ->126A      -0.17439
    122A ->127A      -0.23375
    122A ->128A      -0.11946
    114B ->121B       0.10107
    118B ->121B       0.54314

 Excited State   3:  3.013-A     1.0921 eV 1135.25 nm f=0.0007 <S**2>=2.020
    122A ->124A      -0.64054
    122A ->132A      -0.11947
    111B ->121B       0.13653
    119B ->121B       0.74909
```

Figure S12. TD-DFT/(b3lyp/6-31g(d)) calculation of excited triplet state energies of hexaphenyl isolated molecule at T_1 geometry.

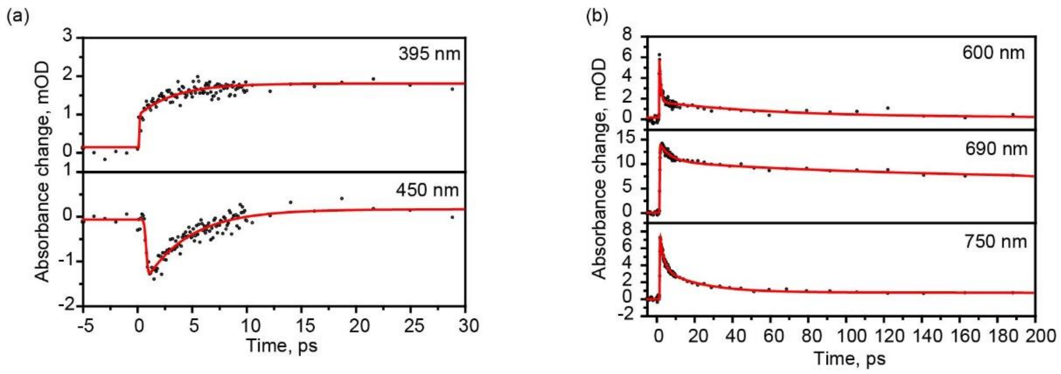

Figure S13. Decay kinetics of the hexaphenyl film λ_{exc} = 250 nm at different wavelengths.

Table S4. Lifetimes obtained from fit/deconvolution of TA spectra for the hexaphenyl film at λ_{exc} = 250 nm; f = fixed.

λ_{exc}, nm	λ_{probe}, nm	τ_1, ps	A_1	τ_2, ps	A_2	τ_3, ps	A_3	τ_4, ps	A_4
250	395	3.4	-0.47					100000^f	1
	450	4.1	-0.74			3300	-0.26	100000^f	1
	600	0.80	0.76	63	0.24				
	690	5.9	0.25	100	0.19	1600	0.39	100000^f	0.17
	750	2.1	0.55	21	0.37	2900	0.08		
